# Weighted Sum-Rate Maximization and Task Completion Time Minimization for Multi-Tag MIMO Symbiotic Radio Networks

**DOI:** 10.3390/s26020644

**Published:** 2026-01-18

**Authors:** Long Suo, Dong Wang, Wenxin Zhou, Xuefei Peng

**Affiliations:** 1 College of Information Science and Technology & Artificial Intelligence, Nanjing Forestry University, Nanjing 210037, China; 8230811052@njfu.edu.cn (D.W.); zwx20240507@163.com (W.Z.); 2 College of Electronics and Information Engineering, Changchun University of Science and Technology, Changchun 130022, China; xfpeng@cust.edu.cn

**Keywords:** symbiotic radio, backscatter communication, weighted sum-rate, weighted minimum mean square error, task completion time, MIMO, resource allocation

## Abstract

Symbiotic radio (SR) has recently emerged as a promising paradigm for enabling spectrum- and energy-efficient massive connectivity in low-power Internet-of-Things (IoT) networks. By allowing passive backscatter devices (BDs) to coexist with active primary link transmissions, SR significantly improves spectrum utilization without requiring dedicated spectrum resources. However, most existing studies on multi-tag multiple-input multiple-output (MIMO) SR systems assume homogeneous traffic demands among BDs and primarily focus on rate-based performance metrics, while neglecting system-level task completion time (TCT) optimization under heterogeneous data requirements. In this paper, we investigate a joint performance optimization framework for a multi-tag MIMO symbiotic radio network. We first formulate a weighted sum-rate (WSR) maximization problem for the secondary backscatter links. The original non-convex WSR maximization problem is transformed into an equivalent weighted minimum mean square error (WMMSE) problem, and then solved by a block coordinate descent (BCD) approach, where the transmit precoding matrix, decoding filters, backscatter reflection coefficients are alternatively optimized. Second, to address the transmission delay imbalance caused by heterogeneous data sizes among BDs, we further propose a rate weight adaptive task TCT minimization scheme, which dynamically updates the rate weight of each BD to minimize the overall TCT. Simulation results demonstrate that the proposed framework significantly improves the WSR of the secondary system without degrading the primary link performance, and achieves substantial TCT reduction in multi-tag heterogeneous traffic scenarios, validating its effectiveness and robustness for MIMO symbiotic radio networks.

## 1. Introduction

The low-power Internet of Things (IoT) has been widely deployed in current 5G systems and is expected to continue to play an essential role in future 6G networks, particularly in industrial IoT, intelligent transportation, smart wearable devices, and smart healthcare applications [1,2]. Due to its inherent advantages, such as reduced manual maintenance requirements and low device-side implementation costs, low-power IoT provides a practical and economically efficient solution for large-scale connectivity [3].

Backscatter communication (BackCom) technology, as a key enabler for achieving low-power and low-cost connectivity, has therefore attracted considerable attention. In BackCom, devices are allowed to transmit their own information by modulating existing radio-frequency (RF) signals in the environment or emitted by other transmitters. By tuning the internal impedance to adjust the reflection coefficient, a Backscatter Device (BD), which is equivalent to a tag in this paper, can vary the amplitude, frequency, or phase of the incident RF signal, thus achieving analog or digital modulation. During this process, the BD does not require any high-power RF front-end components such as transmitters or power amplifiers, nor active RF modules such as Digital-to-Analog Converters or mixers. Thus, a BD’s overall power consumption can be maintained at the microwatt level, orders of magnitude lower than that of traditional wireless devices [4]. Consequently, BackCom can effectively prolong the device lifetime and dramatically reduce system energy consumption and hardware complexity. With the advantages of low cost, ultra-low power and high scalability, BackCom has become a promising solution to massive low-power connectivity for green and sustainable IoT deployments [5].

Currently, BackCom systems are mainly divided into four categories: monostatic backscatter, bistatic backscatter, ambient backscatter, and symbiotic backscatter [6]. Monostatic backscatter originated during World War II, when identification tags were mounted on friendly aircraft to reflect radar signals for recognition. Later, this technology evolved into radio-frequency identification (RFID) systems, which typically consist of a reader and multiple tags, sharing one RF source for both transmission and reception [7,8]. In bistatic backscatter systems, a separate carrier emitter is placed near the tags, which can effectively reduce path loss and extend the communication range. For example, when the carrier power is 20 mW, the tag-to-reader distance can reach about 130 m [9]. Although both monostatic and bistatic systems rely on dedicated RF sources, ambient backscatter systems exploit existing environmental RF signals (e.g., TV or Wi-Fi broadcasts) for communication, achieving even lower power consumption [10,11]. However, in conventional ambient backscatter systems, the passive BackCom link and the active primary communication system are uncoordinated. Uncontrollable environmental RF signals can cause severe interference to the passive link, limiting its performance. To overcome this limitation, a new paradigm—symbiotic backscatter communication, also known as Symbiotic Radio (SR)—was proposed [12,13].

SR enables cooperative transmission between the primary system and the secondary BDs, jointly utilizing the spectrum and the energy of the primary signal. Secondary BDs modulate their information on the RF waveform emitted by the primary transmitter (PT) [13,14,15]. Thus, in SR systems, BDs not only reuse the spectrum as in cognitive radio (CR) systems [16,17,18], but also harvest and reuse the energy of the primary signal through passive backscattering. Moreover, to overcome the poor reliability of conventional ambient backscatter receivers, SR introduces joint reception of the primary and backscattered signals, achieving collaborative decoding and mutual performance enhancement [10,19,20]. Based on the relationship between the symbol durations of the primary and secondary signals, SR can be classified into parasitic SR (PSR) and cooperative SR (CSR, also termed mutualistic SR). In PSR, the symbol durations of the primary and secondary signals are identical, and the secondary transmission interferes with the primary one. In contrast, in CSR, the BD reuses not only the primary spectrum but also its transmit power. The duration of the BD symbol is much longer than that of the primary signal, and the backscattered signal provides additional multipaths that can enhance the primary link. This mutual benefit constitutes the core “symbiosis” of SR [12]. Therefore, SR is a key technology for constructing green, energy-efficient, low-cost, and flexible IoT networks, and is regarded as one of the enabling technologies for ubiquitous intelligent connectivity and green 6G networks [5].

In recent years, extensive theoretical analysis and performance optimization have been conducted on SR systems. For example, the authors of [13] integrated SR with cognitive radio and ambient backscatter for efficient utilization of both spectrum and energy. They proposed receiver and resource-allocation designs for reconfigurable intelligent surface (RIS)-assisted and full-duplex architectures, significantly improving reliability and efficiency. R. Long et al. investigated two beamforming optimization problems, weighted sum-rate (WSR) maximization and transmit power minimization (TPM), in a multiple-input single-output (MISO) SR scenario, solving the non-convex problems via semidefinite relaxation (SDR) [12]. In [21], the mutualistic conditions of CSR in single-input multiple-output (SIMO) channels were examined, and the error-rate and signal-to-noise ratio (SNR) characteristics for various symbol-period ratios *K* were derived. It was proven that secondary transmission can provide multipath gains to the primary link, particularly under high-SNR conditions. X. Kuai et al. proposed a multi-user multi-BD SR receiver in SIMO settings, jointly performing channel estimation, user data decoding, and BD symbol detection. Using factor-graph message passing (MPJE), their design mitigated inter-user and inter-BD interference, reduced pilot overhead, and achieved significant performance gains [22]. The authors in [23] analyzed the multiple-input multiple-output (MIMO) SR scenario and derived achievable rate expressions for active and passive links, revealing their asymptotic relationship as the number of BD increases. The authors further optimized the precoder to maximize the primary rate while ensuring that the secondary rate exceeds a given threshold. Energy efficiency (EE) optimization in SR systems has also attracted research interest. The authors in [24] studied a SISO SR network that jointly optimized PT power, BD reflection coefficients, and TDMA slot durations under both PSR and CSR modes to maximize system EE using a Dinkelbach-based iterative algorithm integrated with block coordinate descent (BCD) and sequential convex programming (SCP). The system achieved the highest EE when the BD with the largest potential gain was assigned the maximum allowed backscatter time while satisfying all throughput constraints. Chu et al. thoroughly investigated resource allocation for optimizing the EE performance of SR systems under the conditions of finite blocklength backscatter links [25]. In [26], cell-free massive MIMO SR frameworks have been proposed to leverage RIS and BackCom to enhance spectral efficiency in IoT networks, highlighting practical trade-offs in hardware complexity and signal processing design between RIS and BD-assisted solutions. Furthermore, the integration of advanced modulation schemes such as orthogonal time-frequency space (OTFS) with SR has been investigated to combat high-mobility channel challenges, illustrating the additional channel estimation and decoding overhead in practical deployments [27].

Despite these advances, most existing SR studies overlook BD heterogeneity, such as variations in payload size and transmission priority, and primarily focus on throughput- or rate-based performance metrics. As a result, the overall system task completion time (TCT)—a critical metric for delay-sensitive and heterogeneous IoT applications—has seldom been investigated. Moreover, the joint design of linear receivers with explicit TCT optimization objectives remains largely under-explored in multi-antenna SR systems. Motivated by these limitations, this paper aims to minimize the system-level TCT in a multi-antenna SR system while explicitly accounting for heterogeneous BDs. To this end, we formulate a TCT-oriented optimization problem that jointly considers BD heterogeneity and linear receiver design. In contrast to existing works that either assume homogeneous BDs or optimize instantaneous rate-based metrics, the proposed approach directly targets task-level performance and provides a unified design framework for heterogeneous SR systems. To clearly position this work with respect to existing studies, Table 1 summarizes the key differences between representative related works and this paper.

The main contributions of this paper are as follows.

- We formulate a WSR maximization problem for a multi-tag MIMO SR system and develop an efficient weight minimum mean square error (WMMSE)-based iterative framework to solve it. Specifically, by exploiting the WSR–WMMSE equivalence, the original non-convex WSR maximization problem is reformulated into a non-convex WMMSE problem, and then solved by the BCD framework, alternatively optimizing the transmit precoding matrix and the backscatter reflection coefficients. With given reflection coefficients, the precoding matrix is obtained from a second-order cone programming (SOCP) problem, and with a given precoding matrix, the reflection coefficients can be derived in closed form.
- We further formulate a TCT minimization problem with the objective of minimizing the maximum TCT of all BDs. To efficiently handle this min–max objective, we design a rate weight adaptive strategy, where the rate weights are iteratively updated according to the heterogeneous transmission times of BDs. By embedding this rate weight adaptive mechanism into the WSR optimization, the proposed method effectively alleviates the bottleneck BD and reduces the overall task completion time without introducing additional scheduling variables.
- Comprehensive numerical results are provided to validate the effectiveness of the proposed framework, demonstrating consistent performance gains in terms of both WSR and TCT when compared with benchmark schemes.

The remainder of this paper is organized as follows. Section 2 introduces the system model of a multi-antenna MIMO SR network and derives the achievable rate expressions for both the primary and secondary links. Section 3 details the WSR maximization problem and the proposed iterative algorithm, incorporating the optimization of BD reflection coefficients. The TCT minimization problem is formulated and solved in Section 4. Numerical simulations and results are discussed in Section 5, followed by concluding remarks in Section 6.

## 2. System Model

**Figure 1:**
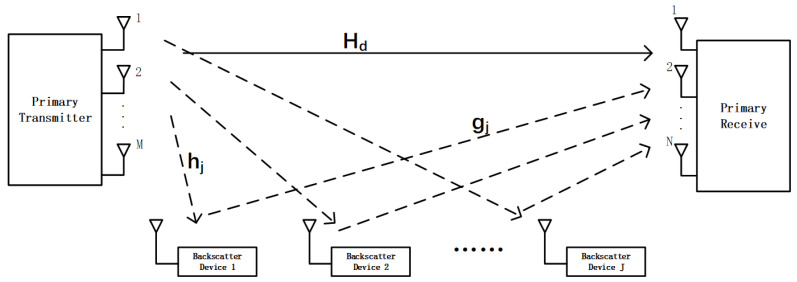
MIMO-SR with multiple BDs.

As illustrated in Figure 1, we consider a multi-tag MIMO SR system. The PT with *N* antennas communicates with a PR equipped with *M* antennas. In addition, *J* single-antenna BDs coexist in the network. Each BD modulates its own information by passively reflecting and modulating the incident RF signal from the PT, thereby achieving simultaneous information transfer and energy harvesting. Consequently, the PR receives and decodes both the primary signal from the PT and the secondary backscattered signals from the *J* BDs. In this symbiotic architecture, the BDs effectively reuse both the spectrum and the radiated power of the primary transmission.

The direct channel from the PT to the PR is denoted by $H_{d}\in C^{M\times N}$. The channel from the PT to the *j*-th BD is $h_{j}\in C^{N\times 1}$, and that from the *j*-th BD to the PR is $g_{j}\in C^{M\times 1}$, for j=1,…,J. Thus, the composite backscatter channel from the PT to the PR via ${\mathrm{BD}}_{j}$ is given by $g_{j}h_{j}^{H}$.

This work focuses on the CSR mode, where the symbol duration of each BD is *K* times longer than that of the PT (K≫1). The complex symbol transmitted by ${\mathrm{BD}}_{j}$ in the *n*-th BD symbol period is $c_{j}\left(n\right)\in C_{BD_{j}}$, satisfying $E[|c_{j}\left(n\right){|}^{2}]=1$. The PT transmits the signal $s\left(k\right)\in C^{d\times 1}$ intended for the PR, where d≤min{M,N} is the number of data streams. The precoding matrix in the PT is $V\in C^{N\times d}$, which is subject to the power constraint $\mathrm{tr}\left(VV^{H}\right)\leq P_{\max}$. The power reflection coefficient of ${\mathrm{BD}}_{j}$ is denoted by $\alpha_{j}\in \left[0,1\right]$. The overall received signal at the PR during the *n*-th BD symbol period can then be written as

(1) $$y\left(k,n\right)=H_{d}Vs\left(k,n\right)+\sum_{j=1}^{J}\sqrt{\alpha_{j}}\;g_{j}h_{j}^{H}Vs\left(k,n\right)c_{j}\left(n\right)+z\left(k,n\right),\;k=1,\ldots ,K.$$

The term $z\left(k,n\right)\in C^{M\times 1}$ denotes an independent and identically distributed (i.i.d.) zero-mean CSCG noise vector, where each entry has variance $\sigma^{2}$, i.e., $z\left(k,n\right)∼\mathrm{CN}\left(0,\sigma^{2}I_{M}\right)$. As the subsequent analysis refers to a fixed interval of BD symbols, the time index *n* is omitted without loss of generality.

We assume that the processing delay introduced by each BD is negligible compared with the symbol duration of the primary transmission. This assumption is commonly adopted in CSR systems, since passive BDs perform instantaneous impedance modulation without baseband processing or active RF chains [28,29,30]. As a result, the direct PT–PR signal and the backscattered signals are assumed to arrive at the PR synchronously within each primary symbol interval. Accordingly, the received signal model simplifies to

(2) $$\begin{array}{c} y\left(k\right)=H_{d}Vs\left(k\right)+\sum_{j=1}^{J}\sqrt{\alpha_{j}}\;g_{j}h_{j}^{H}Vs\left(k\right)c_{j}+z\left(k\right),\;k=1,\ldots ,K. \end{array}$$

Within each period of secondary backscatter symbols, the symbol $c_{j}$ of each ${\mathrm{BD}}_{j}$ is constant. Consequently, the superimposed backscattered signals from all BDs collectively form additional multipath components for the primary link. Defining the vector of the BD symbol as $c={\left[c_{1},c_{2},\ldots ,c_{J}\right]}^{T}$, the equivalent MIMO channel resulting for the primary transmission is given by $H_{\mathrm{eq}}\left(c\right)\in C^{M\times N}$ as

(3) $$H_{\mathrm{eq}}\left(c\right)=H_{d}+\sum_{j=1}^{J}\sqrt{\alpha_{j}}\;g_{j}h_{j}^{H}c_{j}.$$

This equivalent channel $H_{\mathrm{eq}}\left(c\right)$ is a function of the BD symbol vector c. We assume that PR can perfectly estimate $H_{\mathrm{eq}}\left(c\right)$, and that this information is also available at PT through error-free feedback. For a sufficiently large *K*, the average achievable rate for the primary signal is given by

(4) $$R_{s}=E_{c}\;\left[R_{s}\left(c\right)\right]=E_{c}\;\left[\log_{2}\det\;\left(I_{N}+\frac{H_{\mathrm{eq}}\left(c\right)VV^{H}H_{\mathrm{eq}}^{H}\left(c\right)}{\sigma^{2}}\right)\right].$$

In the cooperative symbiotic transmission (CST) mode, the PR employs successive interference cancellation (SIC). It first decodes the primary signal, treating the backscattered signals as interference. After all primary symbols s(k) for k=1,…,K are decoded, the primary signal component (the first term in (2)) is subtracted from the received signal. The residual signal, after cancellation, is given by

(5) $$y_{c}\left(k\right)=\sum_{j=1}^{J}\sqrt{\alpha_{j}}\;g_{j}h_{j}^{H}Vs\left(k\right)c_{j}+z\left(k\right),\;k=1,\ldots ,K.$$

By stacking all *K* received vectors corresponding to one BD symbol period, the expression can be reformulated as

(6) $$Y_{c}=\sum_{j=1}^{J}\sqrt{\alpha_{j}}\;h_{j}g_{j}^{H}VSc_{j}+Z,$$

where

$$Y_{c}=\left[y_{c}\left(k\right),1\leq k\leq K\right]\in C^{M\times K},\;$$

$$S=\left[s(k),1\leq k\leq K\right]\in C^{d\times K},\;$$

and

$$Z=\left[z(k),1\leq k\leq K\right]\in C^{M\times K.}$$

represent, respectively, the secondary received signal matrix within one BD symbol period, the transmitted signal matrix of the PT, and the noise matrix.

To reduce the dimensionality of the received signal matrix, a temporal-domain matched filter $\frac{S^{H}}{\sqrt{K}}\in C^{K\times d}$ is applied, yielding

(7) $${\tilde{Y}}_{c}=\frac{Y_{c}S^{H}}{\sqrt{K}}=\sum_{j=1}^{J}\sqrt{\alpha_{j}}\;h_{j}g_{j}^{H}V\frac{SS^{H}}{\sqrt{K}}c_{j}+\frac{ZS^{H}}{\sqrt{K}}.$$

When *K* is sufficiently large, $\frac{SS^{H}}{K}\approx I_{d}$, and the noise term $\tilde{Z}=\frac{ZS^{H}}{\sqrt{K}}$ can be approximated as an random matrix whose entries follow independent and identical distributions $\mathrm{CN}\;\left(0,\sigma^{2}I_{d}\right)$. Therefore, ${\tilde{Y}}_{c}\in C^{M\times d}$ can be simplified as

(8) $${\tilde{Y}}_{c}=\sum_{j=1}^{J}\sqrt{\alpha_{j}}\;h_{j}g_{j}^{H}Vc_{j}+\tilde{Z}.$$

Furthermore, Equation (8) can be equivalently expressed as a *J*-user uplink multi-user (MU)-MISO channel model, and the corresponding receiving signal is expressed as

(9) $$y_{c}=\sum_{j=1}^{J}h_{c,j}c_{j}+z,$$

where

$$y_{c}=\mathrm{vec}\left({\tilde{Y}}_{c}\right),\;h_{c,j}=\mathrm{vec}\;\left(\sqrt{K\alpha_{j}}\;g_{j}h_{j}^{H}V\right),\;z=\mathrm{vec}\left(\tilde{Z}\right).$$

These are all $N_{d}\times 1$ column vectors, with the noise vector distributed as $z∼\mathrm{CN}(0,\sigma^{2}I_{N_{d}})$.

In this formulation, the system is analogous to an uplink scenario where each BD acts as a single-antenna transmitter and the PR as a multi-antenna receiver with $N_{d}$ effective antennas. Since the effective channel $h_{c,j}$ from ${\mathrm{BD}}_{j}$ to the PR depends on the PT’s precoder V, we employ a linear receiver to decode the secondary signals $c_{j}$ for low-complexity implementation. Successful decoding of all *J* signals requires $J\leq N_{d}$. Each secondary signal $c_{j},1\leq j\leq J$ is detected by using a unit-norm receiving filter $q_{j}\in C^{Nd\times 1}$. The corresponding achievable rate for ${\mathrm{BD}}_{j}$ is given as follows.

(10) $$R_{j}=\log_{2}\left(1+\frac{{\left|q_{j}^{H}h_{c,j}\right|}^{2}}{\sum_{i\neq j}{\left|q_{j}^{H}h_{c,i}\right|}^{2}+\sigma^{2}{∥q_{j}∥}^{2}}\right).$$

We assume block fading channels with perfect channel state information (CSI) are available at both PT and PR [10].

## 3. Weighted Sum-Rate Maximization for BDs

MMSE-based designs have long been used as low-complexity baselines for linear detection/beamforming, typically targeting the minimization of mean square error. Beyond the conventional linear MMSE receiver, the WMMSE framework establishes an equivalence between WSR maximization and weighted MSE minimization, and has become a standard approach for WSR-oriented beamforming in classical multi-user MIMO settings [31]. Iterative extensions, e.g., I-WMMSE, have further enabled distributed or large-scale optimization in interference networks and cell-free massive MIMO systems [32]. Recent modifications have also focused on implementation efficiency, such as matrixless inverters, avoiding the costly real-time deployment of matrix inversion [33]. Different from the above works that mainly optimize conventional primary (or symmetric multi-user) communication links, this paper introduces the WMMSE formulation into the secondary backscatter link of a SR system by explicitly exploiting the PT precoding matrix in the secondary-link effective channel model.

### 3.1. WSR Problem Formulation

The optimization problem of maximizing the WSR for *J* BDs can be formulated as follows.

(11) $$\begin{array}{cc} P1:\;\; & \max_{V,q_{j},\alpha_{j}}\sum_{j=1}^{J}\mu_{j}R_{j}\\ s.t.\; & \mathrm{Tr}\left(VV^{H}\right)\leq P_{\max}\\ \; & 0\leq \alpha_{j}\leq 1,\forall j \end{array}$$

Here, $\mu_{j}$ is a fixed weight for ${\mathrm{BD}}_{j}$. The optimization variables are the receive beamforming vector $q_{j}$, the precoding matrix V, and the reflection coefficients $\alpha_{j}$.

In the WSR problem, the weights $\mu_{j}$ can be adjusted to control individual BD rates. In the CST model, the backscattered signal from each BD can provide multipath diversity to the primary link, potentially improving the signal quality at the PR. However, as the number of BDs increases, the resulting interference among secondary signals may overwhelm the shared communication channel, necessitating a careful balance between the primary rate requirements and the secondary throughput. This balance is enforced by the primary rate constraint in optimization. Generally, accommodating more BDs requires relaxing this primary rate constraint, which also increases the computational complexity of the solution.

The explicit primary-rate constraint is not imposed in P1. According to the primary-rate expression in (4), the primary rate is determined by the precoding matrix V given the effective channel $H_{\mathrm{eq}}$. In solving the WSR maximization problem for secondary links, the local optimal solution $V^{*}$ is usually not optimal for the primary rate. However, deploying more BDs allows the PR to collect more reflected components of the primary signal, which is equivalent to receiving multipath components. Consequently, the effective primary signal power can be enhanced with the help of multiple secondary links. As a result, the achievable rate of the primary link in a multiple-BD scenario is higher than that in a single primary link case, thereby ensuring the primary link rate requirement is automatically met.

The WSR problem is non-convex since the rate expression $R_{j}$ in the objective function is nonconcave in the optimization variables V and $q_{j}$. To address this, we employ the WMMSE approach to equivalently reformulate the problem, denoted as P2 below.

(12) $$\begin{array}{cc} P2:\;\; & \min_{V,\;q_{j},\alpha_{j}}\sum_{j=1}^{J}w_{j}e_{j}\\ s.t.\; & \mathrm{Tr}\;\left(VV^{H}\right)\leq P_{\max}\\ \; & 0\leq \alpha_{j}\leq 1,\forall j \end{array}$$

Here, $e_{j}$ denotes the MSE of the estimated backscatter symbol $c_{j}$, and $w_{j}$ is the corresponding MSE weight. When using a unit-norm receive filter $q_{j}\in C^{N_{d}\times 1}$, the estimated symbol is obtained as

(13) $${\hat{c}}_{j}=q_{j}^{H}y_{c}=q_{j}^{H}h_{c,j}c_{j}+q_{j}^{H}\sum_{i\neq j}^{J}h_{c,i}c_{i}+q_{j}^{H}z.$$

The mean squared error $e_{j}$ is given by

(14) $$e_{j}=E\left[\left({\hat{c}}_{j}-c_{j}\right){\left({\hat{c}}_{j}-c_{j}\right)}^{H}\right]=q_{j}^{H}\Psi_{c}q_{j}-q_{j}^{H}h_{c}^{H}q_{j}-h_{c}^{H}q_{j}+1,$$

where $\Psi_{c}=\sum_{i=1}^{J}h_{c,i}h_{c,i}^{H}+\sigma^{2}I_{N_{d}}$ represents the interference covariance matrix of all BD signals and the noise. To minimize this, the optimal receive filter $q_{j}^{*}$ corresponds to the MMSE receiver, i.e., $q_{j}^{*}=\Psi_{c}^{-1}h_{c,j}$. Thus, the minimum $e_{j}$ can be reformulated as

(15) $$e_{j}=1-h_{c}^{H}q_{j}^{*}h_{c}=\frac{1}{1+h_{c}^{H}{\left(\sum_{i\neq j}h_{c,i}h_{c,i}^{H}+\sigma^{2}I_{N_{d}}\right)}^{-1}h_{c,j}}.$$

By substituting the optimal receiver $q_{j}^{*}=\Psi_{c}^{-1}h_{c,j}$ into (10), one obtains

(16) $$R_{j}=\log_{2}\left(\frac{1}{1-h_{c,j}^{H}\Psi_{c}^{-1}h_{c,j}}\right).$$

Using the Sherman–Morrison–Woodbury matrix identity

$${\left(A+BCD\right)}^{-1}=A^{-1}-A^{-1}B{\left(DA^{-1}B+C^{-1}\right)}^{-1}DA^{-1},$$

we can rewrite $R_{j}$ as

(17) $$\begin{array}{cc} R_{j} & =\log_{2}\left(e_{j}^{-1}\right)=\log_{2}\;\left(1+h_{c,j}^{H}\;{\left(\sum_{i\neq j}^{J}h_{c,i}h_{c,i}^{H}+\sigma^{2}I_{N_{d}}\right)}^{\;-1}\;h_{c,j}\right). \end{array}$$

**Theorem** **1.** *When the precoding matrix V is unitary and the number of transmit antennas equals the number of data streams (i.e., N=d), the achievable rate $R_{j}$ of each BD becomes independent of V.*

**Proof.**  Please refer to Appendix A.    □

It is worth emphasizing that, although $R_{j}$ is a scalar quantity, the above result indicates that it does not depend on the specific realization of the unitary precoder V, rather than implying that it is a universal constant independent of channel realizations or system parameters.

According to Theorem 1, when the number of transmitter antennas and primary data streams are identical, i.e., N=d, it is not necessary to optimize the precoding matrix V. Therefore, the  proposed WSR problem is focused on the case with heterogeneous transmitter and receiver antenna numbers. According to the Karush–Kuhn–Tucker (KKT) conditions, when the weight is set as $w_{j}=\frac{\mu_{j}}{\ln2}e_{j}^{-1}$, the WSR and WMMSE problems share the same stationary points and yield identical objective values at these points [34]. Consequently, maximizing the WSR is equivalent to solving the WMMSE problem P2. Although problem P2 is non-convex in the joint variables $\left(V,q_{j}\right)$ with given $\alpha_{j}$, it is convex with respect to V when $q_{j}$ is fixed, and vice versa. This structure lends itself to an alternating BCD approach, where V, $q_{j}$, and $\alpha_{j}$ are updated alternatively.

### 3.2. Transmit Precoder Optimization

The MSE $e_{j}$ can be expressed in the following second-order cone form when $q_{j}$ and $\alpha_{j}$ are given.

(18) $$\begin{array}{cc} e_{j}\; & =\left(q_{j}^{H}h_{c,j}-1\right){\left(q_{j}^{H}h_{c,j}-1\right)}^{H}+\sum_{i\neq j}q_{j}^{H}h_{c,i}h_{c,i}^{H}q_{j}+\sigma^{2}q_{j}^{H}q_{j}\\ \; & =\left(q_{j}^{H}A_{j}\mathrm{Vec}\left(V\right)-1\right){\left(q_{j}^{H}A_{j}\mathrm{Vec}\left(V\right)-1\right)}^{H}\\ \; & \;+\sum_{i\neq j}q_{j}^{H}\Lambda_{i}\mathrm{Vec}\left(V\right){\mathrm{Vec}}^{H}\left(V\right)\Lambda_{i}^{H}q_{j}+\sigma^{2}q_{j}^{H}q_{j}.\\ \; & ={∥q_{j}^{H}\Omega F-\theta_{j}∥}^{\;2}+\sigma^{2}q_{j}^{H}q_{j}\\ \; & ={∥\mathrm{Vec}(q_{j}^{H}\Omega F-\theta_{j})∥}^{\;2}+\sigma^{2}q_{j}^{H}q_{j}\\ \; & ={∥\left[\begin{array}{c} \sqrt{\sigma^{2}q_{j}^{H}q_{j}}\\ \left(I_{J}⊗q_{j}^{H}\Omega \right)\mathrm{Vec}\left(F\right)-\theta_{j} \end{array}\right]∥}^{2} \end{array}$$

The equivalent vectorized expressions are

$$h_{c,i}=\mathrm{vec}\;\left(\sqrt{K\alpha_{i}}\;g_{i}h_{i}^{H}V\right)=\sqrt{K\alpha_{i}}\;\left(I_{d}⊗g_{i}h_{i}^{H}\right)\mathrm{Vec}\left(V\right)=\Lambda_{i}\mathrm{Vec}\left(V\right),$$

and

$$\;h_{c,j}=\Lambda_{j}\mathrm{Vec}\left(V\right).$$

Here, the (JNd)×J dimension block-diagonal matrix F is represented by

$$F=\mathrm{diag}\;\left(\mathrm{Vec}(V),\;\mathrm{Vec}(V),\;\ldots ,\;\mathrm{Vec}(V)\right)=\left(\begin{array}{cccc} \mathrm{Vec}(V) & 0 & \ldots & 0\\ 0 & \mathrm{Vec}(V) & \ldots & 0\\ \vdots & \vdots & \ddots & \vdots \\ 0 & 0 & \ldots & \mathrm{Vec}(V) \end{array}\right)=I_{J}⊗\mathrm{Vec}\left(V\right).$$

The $N_{d}\times \left(JN_{d}\right)$ dimensional matrix Ω is

$$\begin{array}{c} \Omega =\left[\;\Lambda_{1},\;\Lambda_{2},\;\ldots ,\;\Lambda_{J}\;\right]=\left[\sqrt{K\alpha_{1}}\;I_{J}⊗g_{1}h_{1}^{H},\;\sqrt{K\alpha_{2}}\;I_{J}⊗g_{2}h_{2}^{H},\;\ldots ,\;\sqrt{K\alpha_{J}}\;I_{J}⊗g_{J}h_{J}^{H}\right]. \end{array}$$

The 1×J vector $\theta_{j}$ is

$$\theta_{j}=\left[\begin{array}{c} 0_{1\times (j-1)},\;1,\;0_{1\times (J-j)} \end{array}\right],$$

and ⊗ denotes the Kronecker product operation.

Therefore, when $q_{j}$ and $\alpha_{j}$ are known, the WMMSE problem in (12) can be rewritten in the equivalent form of the following SOCP, from which V can be solved.

(19) $$\begin{array}{cc} P2\text{-}1:\;\min_{V}\; & \sum_{j=1}^{J}l_{j}^{2}\\ s.t.\;\; & ∥\left[\begin{array}{c} \sqrt{w_{j}\sigma^{2}q_{j}^{H}q_{j}}\\ \left[I_{J}⊗\left(w_{j}^{1/2}q_{j}^{H}\Omega \right)\right]\mathrm{Vec}\left(F\right)-w_{j}^{1/2}\theta_{j}^{T} \end{array}\right]∥\leq l_{j},\;j=1,\ldots ,J\\ \; & \mathrm{Tr}\;\left(VV^{H}\right)\leq P_{\max} \end{array}$$

### 3.3. Reflection Coefficient Optimization

Considering the possible impact of the reflection coefficients of the BDs on the overall system performance, the optimization of the reflection coefficient α is incorporated into the WMMSE algorithm framework as follows. Decompose $h_{c,j}$ according to the power reflection coefficient $h_{c,j}=\sqrt{\alpha_{i}}\;{\tilde{h}}_{j}$, where ${\tilde{h}}_{j}=\sqrt{K}\;\left(I_{d}⊗g_{j}h_{j}^{H}\right)\;\mathrm{Vec}\left(V\right)$.

Define $s_{ji}≜|q_{j}^{H}{\tilde{h}}_{i}{|}^{2}$ and fj≜ReqjHh˜j. Thus, with $q_{j}$, $w_{j}$ and V fixed, $e_{j}$ can be written as a function of α as

(20) $$e_{j}\left(\alpha \right)=\sum_{i=1}^{J}s_{ji}\alpha_{i}-2f_{j}\sqrt{\alpha_{j}}+\sigma^{2}{∥q_{j}∥}^{2}+1,\;0<\alpha \leq 1.$$

Then, the weighted MSE can be expressed as

(21) $$\begin{array}{cc} F(\alpha )\; & =\sum_{j=1}^{J}w_{j}e_{j}\left(\alpha \right)\\ \; & =\sum_{j=1}^{J}\left(\sum_{i=1}^{J}w_{j}s_{ji}\right)\alpha_{i}-2w_{j}f_{j}\sqrt{\alpha_{i}}+\mathrm{const}. \end{array}$$

Define $S_{i}≜\sum_{j=1}^{J}w_{j}s_{ji}$ and $g_{i}≜w_{i}f_{i}$, then we have

(22) $$F\left(\alpha \right)=\sum_{i=1}^{J}\phi_{i}\left(\alpha_{i}\right)+\mathrm{const},\;with\;\phi_{i}\left(\alpha \right)=S_{i}\alpha -2g_{i}\sqrt{\alpha }.$$

Here, $\phi_{i}\left(\alpha \right)$ is a convex function. By setting the first-order derivative to zero, the optimal $\alpha_{i}$ can be obtained as

(23) αi*=giSi,αi*=giSi2=wiRe{qiHh˜i}∑j=1Jwj∥qjHh˜i∥22.

The optimal reflection coefficient $\alpha_{i}$ is then calculated for each BD is computed. This optimization of $\left\{\alpha_{j}\right\}$ is integrated into the iterative WMMSE framework, where they are updated after each precoder optimization step for V.

**Theorem** **2.** In the high-SNR regime, the optimal reflection coefficient satisfies $\alpha^{*}\leftarrow \alpha$.

**Proof.**  Please refer to Appendix B.    □

According to the conclusion of Theorem 2, when the transmitted power is relatively high, the optimization steps for $\alpha_{j}$ can also be simplified. However, due to the double-fading effect on the backscatter links, the SR system usually works in the low-to-medium SNR regime.

Thus, the original WSR problem P1 is solved iteratively by addressing a sequence of WMMSE-based subproblems. Following the approach in [23], the algorithm initializes a feasible precoding matrix V and then proceeds iteratively. In each iteration, the weighting factors $w_{j}$, the receiving beamforming vectors $q_{j}$, and the reflection coefficients $\alpha_{j}$ are updated in an alternating fashion. The detailed procedures of the proposed WMMSE-based BD-WSR maximization scheme are presented in Algorithm 1.

The core WMMSE-based iterative framework follows the general methodology developed in [23]. However, in this paper, Algorithm 1 is extended by explicitly incorporating the optimization of the backscatter reflection coefficients, which is tightly coupled with the secondary WSR maximization and the subsequent TCT minimization framework.
**Algorithm 1** Pseudo-code of the BD-WSR maximization Scheme1:Initialize V, $\alpha_{j}=1,1\leq j\leq J$, and ensure $\mathrm{Tr}\left(VV^{H}\right)\leq P_{\max}$.2:**repeat**3:   Update $q_{j}^{\prime }\leftarrow {\left(\sum_{i=1}^{J}x_{i}x_{i}^{H}+\sigma^{2}I\right)}^{-1}x_{j},1\leq j\leq J$;4:   Update $w_{j}^{\prime }\leftarrow \mu_{j}{\left(e_{j}\right)}^{-1},1\leq j\leq J$;5:   Update V by solving the convex SOCP problem (19) with the updated $q_{j}^{\prime }$ and $w_{j}^{\prime }$;6:   Update $\alpha_{j},1\leq j\leq J$ according to Equation (23) with the updated V and $q_{j}^{\prime }$;7:**until** $\left|\sum_{i=1}^{J}\log\left(w_{j}^{\prime }\right)-\sum_{i=1}^{J}\log\left(w_{j}\right)\right|\leq \epsilon$

### 3.4. Complexity Analysis

The computational complexity of the proposed WMMSE algorithm is dominated by the SOCP subproblem in (19). We analyze the computational complexity of one iteration of the proposed WMMSE-based algorithm by decomposing it into three main steps: updating the receive filters $q_{j}$, computing the MSE weights $w_{j}$, and solving the SOCP subproblem.

In the first step, each receive filter $q_{j}$ is obtained via an MMSE-type solution, which requires constructing an MS×MS covariance matrix, performing a matrix inversion (or equivalently a Cholesky decomposition), and multiplying the inverse with a vector. The dominant operation is the matrix inversion, resulting in a per-$q_{j}$ computational complexity of $O({\left(MS\right)}^{3})$. Updating all *J* receive filters therefore incurs a total complexity of $O(J{\left(MS\right)}^{3})$.

In the second step, the MSE weight $w_{j}$ is computed from the instantaneous MSE $e_{j}$, which involves several inner products of MS-dimensional vectors and one quadratic form, dominated by a matrix–vector multiplication of size MS×MS. Hence, the complexity of computing one $w_{j}$ is $O({\left(MS\right)}^{2})$, and updating all weights results in a total complexity of $O(J{\left(MS\right)}^{2})$.

In the third step, the beamforming update is formulated as a second-order cone programming (SOCP) problem with O(MS) real optimization variables, O(J) second-order cone constraints, and each cone having dimension O(MS). By employing a standard interior-point method [35], the worst-case complexity of solving the SOCP is given by $O\;\left(J^{1.5}{\left(MS\right)}^{3}\log\;\left(\frac{1}{\varepsilon }\right)\right),$ where ε denotes the solution accuracy. By combining the above results, the overall computational complexity per WMMSE iteration is $O\;\left(J{\left(MS\right)}^{3}+J{\left(MS\right)}^{2}+J^{1.5}{\left(MS\right)}^{3}\log\;\left(\frac{1}{\varepsilon }\right)\right).$ Since the SOCP term grows faster with respect to both *J* and MS, it dominates the total computational cost. Therefore, the overall per-iteration complexity of the proposed algorithm scales as $O\;\left(J^{1.5}{\left(MS\right)}^{3}\log\;\left(\frac{1}{\varepsilon }\right)\right).$

## 4. TCT Minimization for Multi-BD SR

Although TCT minimization has been extensively investigated in other research domains, such as job and flow scheduling in cluster computing [36,37,38], its integration with multi-tag MIMO SR networks and WMMSE-based joint resource allocation has not been thoroughly explored. Different from scheduling-oriented formulations that typically introduce explicit scheduling variables such as job ordering, migration, or coflow-level constraints, the proposed approach embeds task-awareness directly into the WSR optimization framework by iteratively adapting the rate weights according to the heterogeneous transmission times of BDs. This strategy avoids introducing additional scheduling variables and maintains the tractable WMMSE/SOCP structure, enabling efficient joint optimization of the transmit beamforming, receive beamforming, and reflection coefficients while effectively reducing the minimum TCT.

We now address the TCT minimization problem. Let $D_{j}$ denote the data size required by ${\mathrm{BD}}_{j}$. The time needed to transmit this data at rate $R_{j}$ is given by

(24) $$T_{j}=\frac{D_{j}}{R_{j}}.$$

Thus, the TCT optimization problem can be formulated as follows.

(25) $$\begin{array}{cc} P3:\;\; & \min_{V,\alpha_{j}}\left\{\max\left(T_{j}\right)\right\}\\ s.t.\;\; & \mathrm{Tr}\;\left(VV^{H}\right)\leq P_{\max},\forall j\\ \; & R_{j}\leq R_{\max},\forall j\\ \; & T_{j}\leq T_{\max},\forall \;j\\ \; & 0\leq \alpha_{j}\leq 1,\forall j \end{array}$$

A weight-adaptation strategy is proposed to minimize TCT. In this strategy, the optimization weights $\mu_{j}$ are dynamically assigned according to the data size $D_{j}$ of each BD. This adjustment aims to balance the transmission load among the BDs, which mitigates bottleneck effects and minimizes the overall TCT.

The weight for the *j*-th BD, denoted μ(j), is defined as

(26) $$\mu_{j}=\frac{T_{j}}{\frac{1}{J}\sum_{j=1}^{J}T_{j}}.$$

With the updated weights μ(j), the WSR problem is re-optimized to obtain the new transmission rates $R_{j}$ for all BDs. This weight-adjustment process iterates until the rates converge, i.e., the deviation among all $R_{j}$ falls below a predefined threshold. The corresponding transmission time for each BD is then given by

(27) $$T_{j}^{\prime }=\frac{D_{j}}{R_{j}^{\prime }}.$$

The detailed procedures of the proposed rate weight adaptive TCT minimization scheme are presented in Algorithm 2.
**Algorithm 2** Pseudo-code of the rate weight adaptive TCT minimization Scheme1:Use Algorithm 1 to compute the initial rate $R_{j}$ for each BD.2:Compute each BD’s transmission time $T_{j}=D_{j}/R_{j}$.3:**repeat**4:   Update weights for all BDs, $\mu_{j}=\frac{T_{j}}{\frac{1}{J}\sum_{j=1}^{J}T_{j}},1\leq j\leq J$.5:   **repeat**6:     Update $q_{j}^{\prime }\leftarrow {\left(\sum_{i=1}^{J}x_{i}x_{i}^{H}+\sigma^{2}I\right)}^{-1}x_{j},1\leq j\leq J$;7:     Update $w_{j}^{\prime }\leftarrow \mu_{j}^{\prime }{\left(e_{j}\right)}^{-1},1\leq j\leq J$;8:    Update V by solving the convex SOCP problem (19) with the updated $q_{j}^{\prime }$ and $w_{j}^{\prime }$;9:    Update $\alpha_{j},1\leq j\leq J$ according to Equation (23) with the updated V and $q_{j}^{\prime }$;10:   **until** $\left|\sum_{i=1}^{J}\log\left(w_{j}^{\prime \prime }\right)-\sum_{i=1}^{J}\log\left(w_{j}^{\prime }\right)\right|\leq \epsilon$11:   Recompute $R_{j}$ based on the updated $V^{\prime }$ and $\alpha_{j}$, and then update $T_{j}=D_{j}/R_{j}$.12:**until** $T_{\max}-T_{\min}\leq \epsilon^{\prime }$

## 5. Simulation and Results

In this section, we numerically evaluate the performance of the proposed BD-WSR maximization scheme and the rate weight adaptive TCT minimization scheme. All simulations are conducted under independent Rayleigh fading channels. The noise power is identical for all users. The transmit power on each transmitter is set to $P=10^{\frac{SNR}{10}}$, and SNR ranges from 5 to 30 dB. In addition, the symbol-duration ratio between the BD symbols and the PT symbols is fixed at K=128. Unless otherwise specified, all results are averaged over 500 Monte Carlo trials. We examine two representive scenarios: Scenario A with N=M=d=3, and Scenario B with N=6 and d=M=3. We begin by comparing the system performance with perfect CSI, and then proceed to consider the scenarios with both imperfect CSI and imperfect SIC.

**Figure 2:**
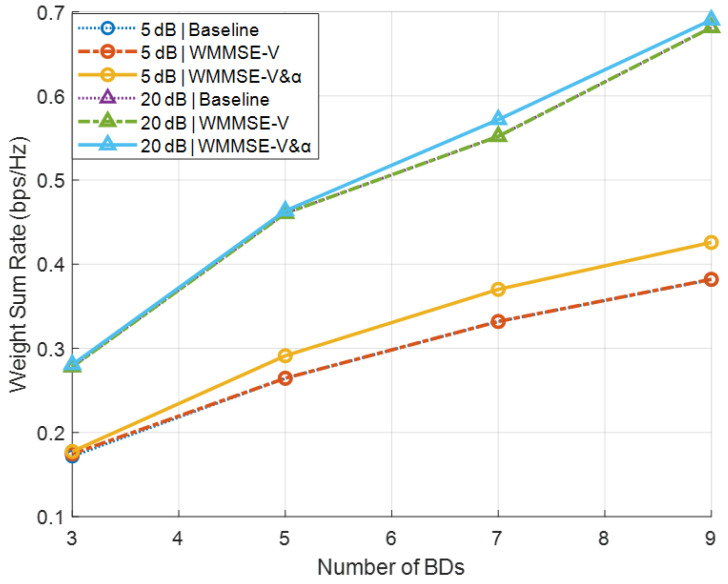
Secondary sum-rate versus the number of BDs *J* in Scenario A.

Figure 2 plots the secondary sum-rate versus the number of BDs *J* for the baseline, WMMSE, and α-update schemes under low-SNR (5dB) and high-SNR (20dB) conditions in Secnario A. The baseline scheme is included to verify the theoretical observation in Theorem 1, which indicates that when the precoder is unitary, the secondary rate is invariant with respect to V. Therefore, the overlapping curves confirm the analytical result rather than diminishing the effectiveness of the proposed method. In the baseline scheme, the precoder is initialized as a random unitary matrix V, and each reflection coefficient $\alpha_{j}$ is drawn randomly from [0, 1]. To isolate the algorithmic contribution, both the WMMSE and α-update schemes are warm-started from the same baseline initialization, and all schemes are simulated over identical Rayleigh channel realizations. The results indicate that optimizing V via WMMSE alone has a negligible effect on the secondary sum-rate. This is mainly because the BD rate becomes constant when V is unitary, as shown in Theorem 1. Enabling the α update within the WMMSE pipeline yields noticeable gains at low SNR, though the improvement is marginal at high SNR. This behavior occurs because, when M=N and V is unitary, the α-update mapping approximates an identity function, causing its output to remain close to its input (see Theorem 2).

**Figure 3:**
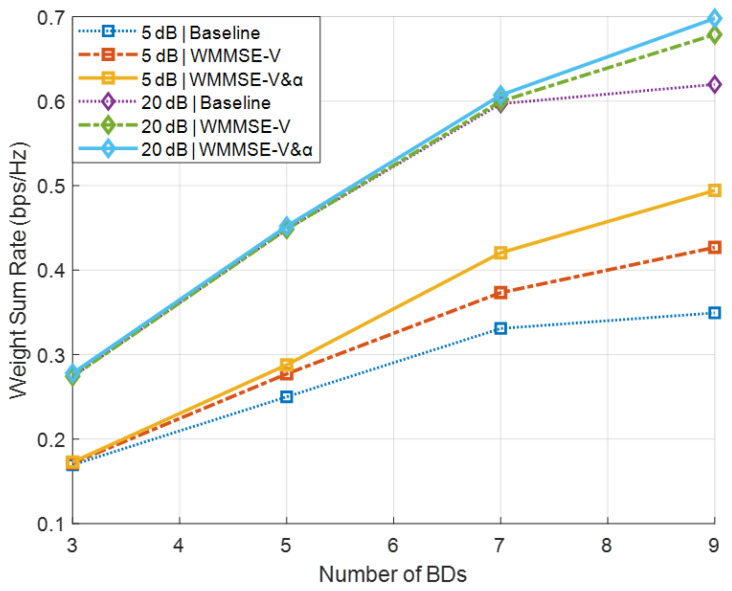
Secondary sum-rate versus the number of BDs *J* in Scenario B.

Figure 3 shows the corresponding results for Scenario B, where the precoder V is initialized as column-orthogonal and other settings remain the same as in Scenario A. In this case, both the WMMSE method and the α-update scheme provide significant secondary sum-rate improvements under both low- and high-SNR conditions. Although Figure 2 and Figure 3 share a similar presentation, they correspond to fundamentally different antenna configurations. Specifically, Figure 2 represents the special case N=d, where the precoder optimization has limited impact, whereas Figure 3 illustrates the general case N>d, in which the proposed WMMSE-based design fully exploits the spatial degrees of freedom.

**Figure 4:**
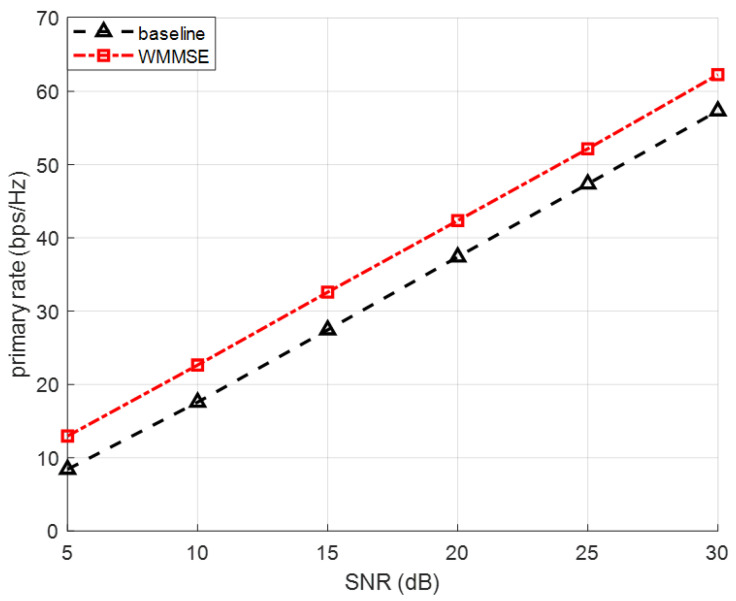
Primary Rate Performance under Different SNR levels.

Figure 4 illustrates the achievable primary-link rate versus SNR for the case of N=6, M=d=3 with a fixed number of BDs J=9. The curves compare the baseline scenario without any BDs and the proposed WMMSE-based optimization framework, where both the precoder *V* and the reflection coefficients α are optimized jointly. As shown in the figure, the primary rate monotonically increases with SNR in both cases. More importantly, the presence of BDs does not introduce any degradation to the primary link, even when nine BDs are simultaneously backscattering. This confirms the inherent property of the CSR model, where each BD contributes an additional effective multipath component to the primary PT–PR channel. After WMMSE-based optimization, the precoder and reflection coefficients adaptively balance the useful enhancement and potential interference, ensuring that the primary-link performance is preserved. Throughout the SNR range (5–30 dB), the optimized case assisted by BD achieves nearly identical primary rates to the baseline without BD, demonstrating that the proposed optimization framework successfully prevents interference leakage from the secondary backscatter link. These results validate that introducing multiple BDs and optimizing their parameters does not create negative effects on the primary transmission, while still enabling significant improvements in the secondary system performance.

**Figure 5:**
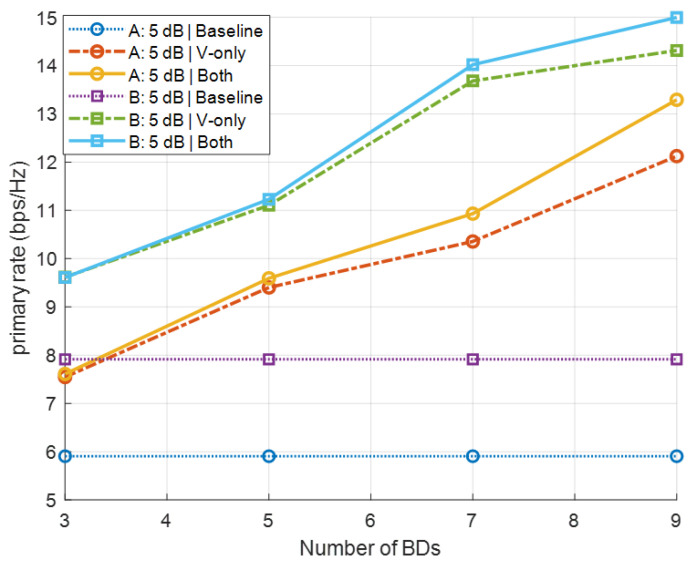
Primary rate versus the number of BDs *J* under Scenarios A and B.

Figure 5 shows the primary rate versus the number of BDs J∈{3,5,7,9} with the SNR of 5 dB for Scenarios A and B. For each scenario, three schemes are compared: (i) a baseline without any BDs, (ii) a BD-assisted scheme where only the precoder V is optimized via WMMSE, and (iii) the proposed BD-assisted scheme with joint WMMSE optimization of V and the reflection coefficients α. The numerical results demonstrate that the proposed joint optimization yields a significant performance gain. In particular, optimizing only the precoder V provides a marginal improvement over the baseline, whereas the joint optimization of both V and α achieves the highest primary-link rate among all the schemes.

**Figure 6:**
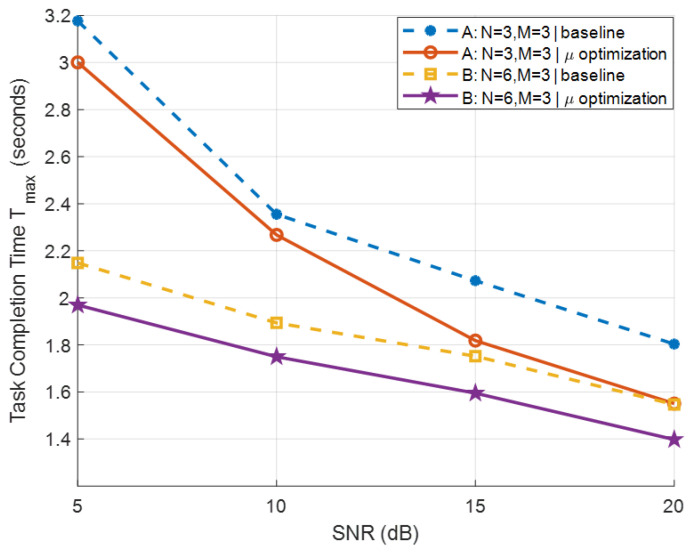
TCT performance versus SNR under fixed and adaptive rate weight schemes.

Next, we evaluate the proposed rate weight adaptive TCT minimization scheme. The payload size for each BD is randomly sampled from the range [0.5,1.5]KB, and the system bandwidth is fixed at $10^{4}\;\mathrm{Hz}$. Figure 6 presents the TCT performance comparison between the proposed rate weight adaptive method and a baseline with fixed rate weights $w_{j}=1,\forall j$. The results demonstrate that across various SNR levels and antenna configurations (M,N), the proposed design consistently yields a substantial reduction in TCT, thereby enhancing overall system efficiency.

**Figure 7:**
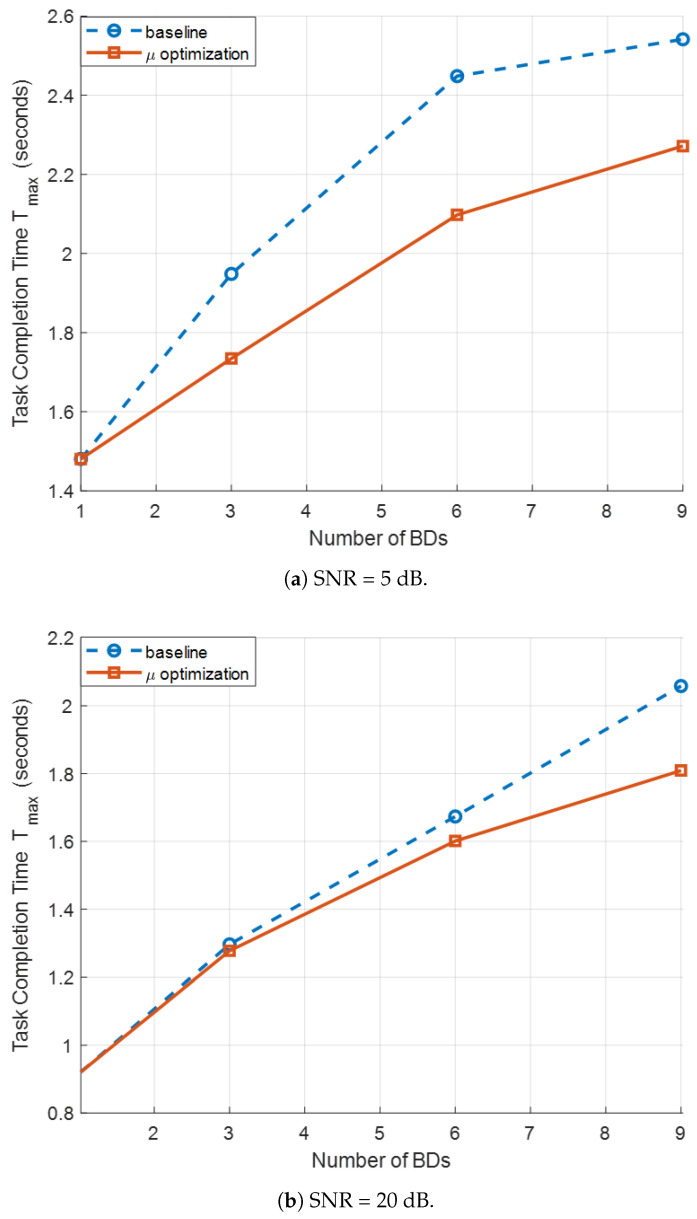
TCT comparison of fixed rate weights and adaptive rate weights for different BD numbers.

Figure 7a,b illustrate the TCT results versus the number of BDs, J∈{1,3,6,9}, for SNR values of 5 dB and 20 dB, respectively, under the MIMO SR configuration with N=6 and M=S=3. Two schemes are compared: (i) a baseline scheme with fixed rate optimization weights $\mu_{j}$, and (ii) the proposed rate weight adaptive strategy, where the weights $\mu_{j}$ are iteratively updated based on each device’s current transmission time $T_{j}=D_{j}/R_{j}$, as detailed in Algorithm 2. As *J* increases, the baseline scheme shows a sharp increase in the maximum TCT. This occurs because BDs with relatively low achievable rates become bottlenecks, dominating the overall completion time. In contrast, the proposed weight adaptive method significantly reduces the TCT for all considered *J*. By assigning larger weights to BDs with longer transmission times, the WMMSE-based resource allocation balances the completion times across devices. This results in a more balanced rate distribution and a substantial reduction in the maximum $T_{j}$. The performance gain is particularly evident with a large number of BD (e.g., J=6 and 9), where the competition for the shared symbiotic link intensifies. These results indicate that intelligently adapting $\mu_{j}$ is crucial to minimize TCT and improve system-level efficiency in MIMO SR systems. The fixed-weight baseline fails to fully exploit the available degrees of freedom in the precoder and reflection-coefficient design. Moreover, the optimization gain is more pronounced at low SNR than at high SNR. This can be attributed to the inner loop of Algorithm 2, which relies on WMMSE iterations whose impact is stronger in noise-limited regimes.

**Figure 8:**
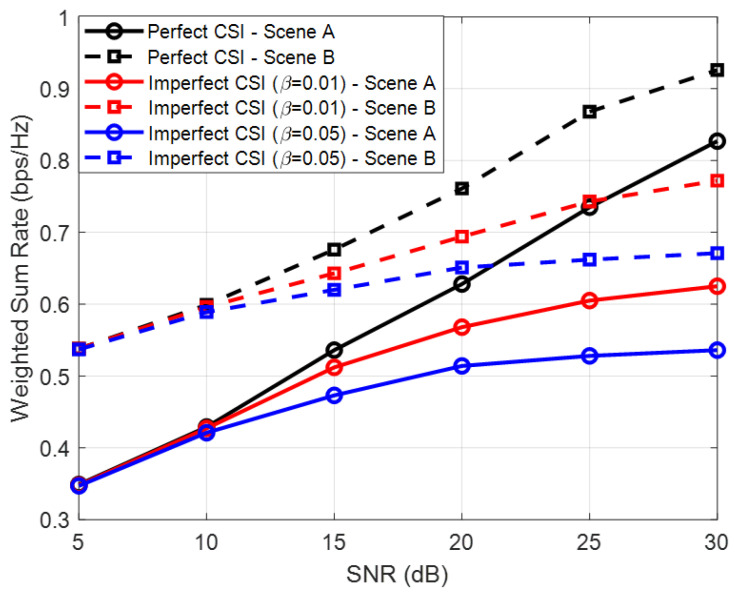
BD-WSR versus SNR under perfect and imperfect CSI of the equivalent channel.

To evaluate the robustness of the proposed design under practical channel uncertainty, we further consider an imperfect CSI scenario for the equivalent channel. The imperfect CSI is modeled by a correlated additive error model, where the estimated equivalent channel is given by $\hat{h}=\sqrt{1-\beta }\;h+\sqrt{\beta }\;e,$ in which *h* denotes the true equivalent channel, i.e., $g_{j}$ or $h_{j}$, *e* represents the additive noise vector with i.i.d. circularly symmetric complex Gaussian entries, and β∈[0,1] characterizes the level of CSI imperfection, with β=0 corresponding to the perfect CSI case. As shown in Figure 8, the achievable WSR under imperfect CSI is lower than that under perfect CSI, and the performance degradation becomes more noticeable as the CSI error level β increases. The proposed optimized scheme exhibits significant performance degradation with respect to CSI errors. Even with low CSI imperfection levels, e.g., β=0.01, the overall increasing trend of the BD’s WSR with respect to SNR is no longer preserved. In the high-SNR regime, the WSR under imperfect CSI gradually approaches a saturation level, since the residual CSI errors become the dominant performance-limiting factor and cannot be mitigated by further increasing the transmit power. This indicates that the proposed design is less robust to moderate CSI imperfections and remains less effective in high SNR scenarios where perfect CSI cannot be guaranteed.

In the previous analysis, ideal SIC is assumed, where the primary signal can be perfectly removed before detecting the BDs. In practice, however, SIC may be imperfect due to channel estimation errors, noise enhancement, and error propagation, which leads to residual primary signal interference. Let $\hat{s}\left(k\right)$ denote the estimated primary signal and define the estimation error as $\Delta s\left(k\right)=s\left(k\right)-\hat{s}\left(k\right)$. After SIC, the received signal for BD detection can be written as

$$y_{c}^{\mathrm{imp}}\left(k\right)=\sum_{j=1}^{J}\sqrt{\alpha_{j}}g_{j}h_{j}^{H}Vs\left(k\right)c_{j}+H_{d}V\Delta s\left(k\right)+z\left(k\right),$$

where the second term represents the residual primary signal caused by imperfect SIC. Following standard practice, the estimation error is modeled as a zero-mean complex Gaussian random vector with covariance

$$E\left\{\Delta s\left(k\right)\Delta s^{H}\left(k\right)\right\}=\epsilon_{s}I,$$

where $\epsilon_{s}\in \left[0,1\right]$ denotes the SIC imperfection factor. The ideal SIC case is recovered when $\epsilon_{s}=0$. Accordingly, the interference-plus-noise covariance matrix for BD detection becomes

$$\Psi_{c}^{\mathrm{imp}}=\sum_{i=1}^{J}h_{c,i}h_{c,i}^{H}+\sigma^{2}I+\epsilon_{s}B_{s},$$

where

$$B_{s}=I_{M}⊗\left(H_{d}VV^{H}H_{d}^{H}\right).$$

The resulting SINR for the *j*-th BD is given by

$${\mathrm{SINR}}_{j}^{\mathrm{imp}}=\frac{|q_{j}^{H}h_{c,j}{|}^{2}}{\sum_{i\neq j}|q_{j}^{H}h_{c,i}{|}^{2}+\sigma^{2}{∥q_{j}∥}^{2}+\epsilon_{s}q_{j}^{H}B_{s}q_{j}},$$

and the corresponding achievable rate is

$$R_{j}^{\mathrm{imp}}=\log_{2}\left(1+{\mathrm{SINR}}_{j}^{\mathrm{imp}}\right).$$

**Figure 9:**
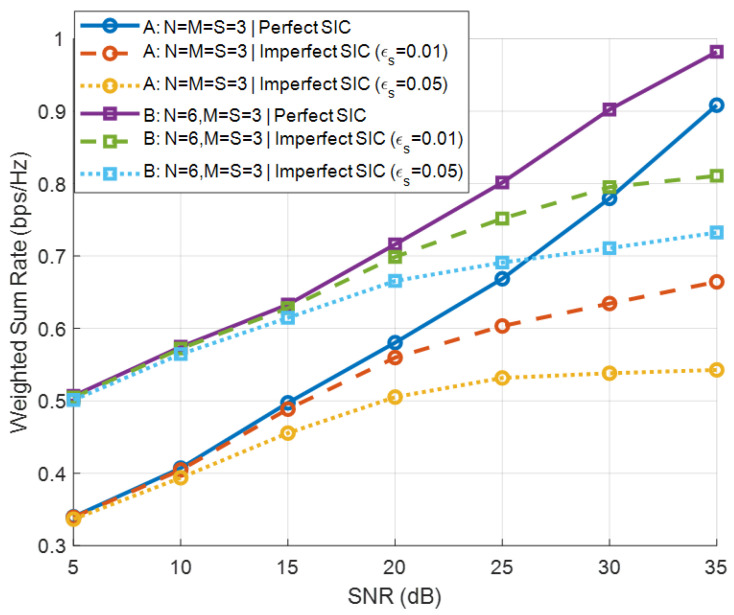
BD-WSR versus SNR under perfect and imperfect SIC.

Figure 9 illustrates the BD-WSR performance under perfect SIC and imperfect SIC with $\epsilon_{s}=0.01$ and $\epsilon_{s}=0.05$. It is observed that imperfect SIC results in rate degradation compared with the ideal case, and the performance gap increases with SNR due to the stronger residual primary interference. Nevertheless, the proposed optimization framework remains effective, and the performance loss is moderate, demonstrating the robustness of the proposed design against SIC imperfections.

## 6. Conclusions

This paper investigates a multiple-BD MIMO SR system. We formulate the weighted secondary sum-rate maximization problem and solve it via a WMMSE-based BCD framework, alternatively optimizing the precoding matrix and reflection coefficients. Moreover, to minimize the overall TCT of all BDs, we propose a rate weight adaptive TCT minimization scheme that dynamically adjusts the specific rate weight of each BD. Numerical simulation results show that this scheme significantly enhances the WSR and reduces the TCT of BDs. Future work will extend the proposed framework to multi-user primary networks, incorporate more realistic channel models, and investigate hardware implementation considerations for practical SR systems.

## Figures and Tables

**Table 1:** Comparison between representative related works and this paper.

Work	Multi-Tag SR	MIMO	BD Heterogeneity	TCT-Aware Design
Long et al. [12]	×	×	×	×
Chen et al. [28]	✓	×	×	×
Kuai et al. [22]	✓	✓	×	×
Xu et al. [23]	✓	✓	×	×
This work	✓	✓	✓	✓

## Data Availability

The original contributions presented in this study are included in the article. Further inquiries can be directed to the corresponding author.

## Appendix A. Proof of Constant Determinant Property

**Proof.** We aim to prove that

$$\det\;\left(I_{K}+{\left(\sum_{k}h_{k}h_{k}^{H}+\sigma^{2}I_{K}\right)}^{-1}h_{1}h_{1}^{H}\right).$$

is a constant, where $h_{i}=\mathrm{vec}\left(A_{i}V\right)$, and V is a unitary matrix. Using the matrix determinant lemma,

$$\det\left(I+B^{-1}h_{1}h_{1}^{H}\right)=1+h_{1}^{H}B^{-1}h_{1},$$

where

$$B=\sum_{k}h_{k}h_{k}^{H}+\sigma^{2}I_{K}.$$

Hence, it suffices to show that $h_{1}^{H}B^{-1}h_{1}$ is constant. Let $H=[h_{2},h_{3},\ldots ,h_{K}]$; then B can be rewritten as

$$B=\sigma^{2}I+HH^{H}.$$

Using the Woodbury matrix identity,

$${\left(A+UV\right)}^{-1}=A^{-1}-A^{-1}U{\left(I+VA^{-1}U\right)}^{-1}VA^{-1}.$$

Let $A=\sigma^{2}I$, U=H, and $V=H^{H}$. Then

$$B^{-1}=\frac{1}{\sigma^{2}}I-\frac{1}{\sigma^{4}}H{\left(I+\frac{1}{\sigma^{2}}H^{H}H\right)}^{-1}H^{H}.$$

Hence,

$$h_{1}^{H}B^{-1}h_{1}=\frac{1}{\sigma^{2}}h_{1}^{H}h_{1}-\frac{1}{\sigma^{4}}h_{1}^{H}H{\left(I+\frac{1}{\sigma^{2}}H^{H}H\right)}^{-1}H^{H}h_{1}.$$

Note that $h_{i}=\mathrm{vec}\left(A_{i}V\right)$. Using the vectorization property,

$$h_{i}^{H}h_{j}=\mathrm{vec}{\left(A_{i}V\right)}^{H}\mathrm{vec}\left(A_{j}V\right)=\mathrm{tr}\;\left(A_{i}^{H}VA_{j}V^{H}\right)=\mathrm{tr}\;\left(V^{H}A_{i}^{H}A_{j}V\right).$$

Since V is unitary, $VV^{H}=I$, we have

$$\mathrm{tr}\;\left(V^{H}A_{i}^{H}A_{j}V\right)=\mathrm{tr}\;\left(A_{i}^{H}A_{j}\right).$$

Therefore, $h_{i}^{H}h_{j}=\mathrm{tr}\left(A_{i}^{H}A_{j}\right)$ is constant. The elements of $H^{H}H$ are

$${\left(H^{H}H\right)}_{ij}=h_{i}^{H}h_{j}=\mathrm{tr}\left(A_{i}^{H}A_{j}\right),$$

which are all constants. Thus, $H^{H}H$ is constant, and consequently,

$$h_{1}^{H}H{\left(I+\frac{1}{\sigma^{2}}H^{H}H\right)}^{-1}H^{H}h_{1}.$$

is also constant. Therefore,

$$\det\left(I+B^{-1}h_{1}h_{1}^{H}\right)=1+h_{1}^{H}B^{-1}h_{1}.$$

is a constant. □

## Appendix B. Asymptotic Property of the Reflection Coefficient Update

**Proof.** Let

$$\begin{array}{cc} H & =\left[h_{c,1},\ldots ,h_{c,J}\right]=\left[\sqrt{\alpha_{1}}h_{1},\ldots ,\sqrt{\alpha_{J}}h_{J}\right],\\ A & =HH^{H}=\sum_{k=1}^{J}h_{c,k}h_{c,k}^{H}. \end{array}$$

When the SNR is high, the MMSE receiver can be approximated as

$$q_{j}=A^{-1}h_{c,j}.$$

When V is a full-rank matrix, the following identity holds:

$$H^{H}A^{-1}H=I_{J}.$$

Expanding the *i*-th element gives

$$q_{j}^{H}h_{c,i}=h_{c,j}^{H}A^{-1}h_{c,i}=\delta_{ji},\;\mathrm{where}\;\delta_{ji}=\left\{\begin{array}{cc} 1, & j=i,\\ 0, & j\neq i. \end{array}\right.$$

Since $h_{c,i}=\sqrt{\alpha_{i}}h_{i}$, we have

$$q_{j}^{H}h_{i}=\frac{\delta_{ji}}{\sqrt{\alpha_{i}}},\;q_{i}^{H}h_{i}=\frac{1}{\sqrt{\alpha_{i}}}.$$

Define

Si=∑j=1Jwj|qjHhi|2=∑j=1Jwjδji2αi=wiαi,gi=wiRe{qiHhi}=wiαi.

Then,

$$\frac{g_{i}}{S_{i}}=\sqrt{\alpha_{i}}.$$

The optimal update is therefore

$$t_{i}^{*}=\frac{g_{i}}{S_{i}}\Rightarrow \alpha_{i}^{\mathrm{new}}=\alpha_{i}.$$

This proves that, under high-SNR conditions, the reflection coefficient $\alpha_{i}$ returns to its original value after the update. When $\sigma^{2}>0$ but remains relatively small, we have

$$q_{i}={\left(A+\sigma^{2}I\right)}^{-1}h_{c,i}.$$

Using the first-order expansion

$${\left(A+\sigma^{2}I\right)}^{-1}=A^{-1}-\sigma^{2}A^{-2}+O\left(\sigma^{4}\right),$$

we obtain

$$\begin{array}{cc} H^{H}{\left(A+\sigma^{2}I\right)}^{-1}H & =H^{H}A^{-1}H-\sigma^{2}\;H^{H}A^{-2}H+O\left(\sigma^{4}\right)\\ \; & =I-\sigma^{2}\Gamma +O\left(\sigma^{4}\right), \end{array}$$

where

$$\Gamma =H^{H}A^{-2}H=H^{H}{\left(HH^{H}\right)}^{-2}H,$$

which is a J×J positive semidefinite Hermitian matrix independent of $\sigma^{2}$. It serves as a correction term for the high-SNR approximation. Using the above expression, we obtain

$$q_{i}^{H}h_{i}=\frac{\delta_{ji}}{\sqrt{\alpha_{i}}}+O\left(\sigma^{2}\right),$$

and therefore

$$S_{i}=\frac{w_{i}}{\alpha_{i}}+O\left(\sigma^{2}\right),\;g_{i}=\frac{w_{i}}{\sqrt{\alpha_{i}}}+O\left(\sigma^{2}\right),\;\frac{g_{i}}{S_{i}}=\sqrt{\alpha_{i}}+O\left(\sigma^{2}\right).$$

Hence, the update error is of order $O(\sigma^{2})$. As the SNR increases, the deviation decreases. □
